# Grand Rounds: An Outbreak of Toxic Hepatitis among Industrial Waste Disposal Workers

**DOI:** 10.1289/ehp.8951

**Published:** 2006-09-18

**Authors:** Hae-Kwan Cheong, Eun A Kim, Jung-Keun Choi, Sung-Bong Choi, Jeong-Ill Suh, Dae Seob Choi, Jung Ran Kim

**Affiliations:** 1 Department of Social and Preventive Medicine, Sungkyunkwan University School of Medicine, Suwon, Korea; 2 Occupational Safety and Health Research Institute, Korea Occupational Safety and Health Agency, Incheon, Korea; 3 Department of Internal Medicine, College of Medicine, Dongguk University, Gyeongju, Korea; 4 Department of Diagnostic Radiology, College of Medicine, Gyeongsang National University, Jinju, Korea; 5 Department of Pathology, College of Medicine, Dongguk University, Gyeongju, Korea

**Keywords:** complex exposure, dimethylacetamide, hepatotoxicity, industrial waste, liver biopsy, toxic hepatitis

## Abstract

**Context:**

Industrial waste (which is composed of various toxic chemicals), changes to the disposal process, and addition of chemicals should all be monitored and controlled carefully in the industrial waste industry to reduce the health hazard to workers.

**Case presentation:**

Five workers in an industrial waste plant developed acute toxic hepatitis, one of whom died after 3 months due to fulminant hepatitis. In the plant, we detected several chemicals with hepatotoxic potential, including pyridine, dimethylformamide, dimethylacetamide, and methylenedianiline. The workers had been working in the high-vapor-generating area of the plant, and the findings of pathologic examination showed typical features of acute toxic hepatitis.

**Discussion:**

Infectious hepatitis and drug-induced hepatitis were excluded by laboratory findings, as well as the clinical course of hepatitis. All cases of toxic hepatitis in this plant developed after the change of the disposal process to thermochemical reaction–type treatment using unslaked lime reacted with industrial wastes. During this chemical reaction, vapor containing several toxic materials was generated. Although we could not confirm the definitive causative chemical, we suspect that these cases of hepatitis were caused by one of the hepatotoxic agents or by a synergistic interaction among several of them.

**Relevance to clinical or professional practice:**

In the industrial waste treatment process, the danger of developing toxic hepatitis should be kept in mind, because any subtle change of the treatment process can generate various toxic materials and threaten the workers’ health. A mixture of hepatotoxic chemicals can induce clinical manifestations that are quite different from those predicted by the toxic property of a single agent.

## Case Presentation

From May to September 2001, two women and three men working in an industrial waste treatment plant in Ulsan, Korea, developed acute hepatitis.

**Figure 1:**
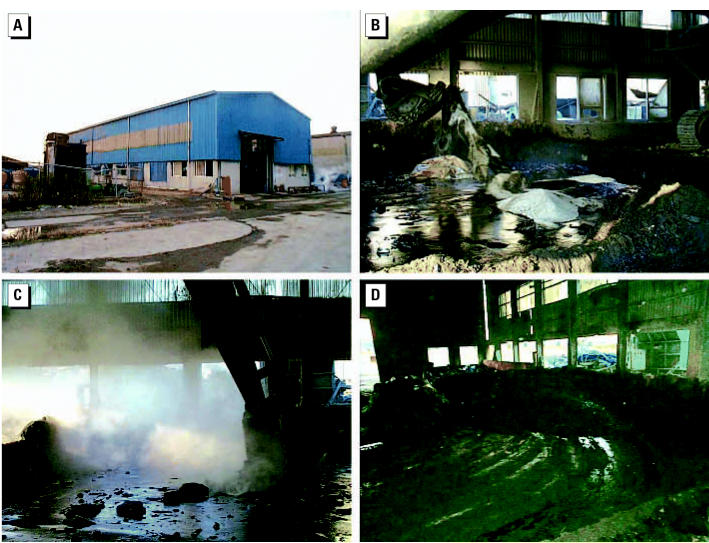
Process of unslaked lime solidification reaction. (*A*) Reaction is performed in a warehouse with the windows removed. (*B*) For each reaction, 100–250 drums of liquid wastes and 7–12 tons of unslaked lime are loaded in a constant ratio with liquid wastes. (*C*) As the wastes and unslaked lime are mixed, thick noxious vapors rise during the heat reaction and escape through the open windows; the full reaction takes > 12 hr with emission of huge amounts of noxious gases and vapors. (*D*) Final stage of the process after 10 hr of thermochemical reaction.

To more efficiently treat a larger quantity of waste, in May 2000 the plant introduced a new process solidifying liquid wastes through thermochemical reaction evoked by unslaked lime, thereby evaporating volatiles. During this process, workers put 100–250 drums (200 L) of liquid wastes and 7–12 tons of unslaked lime into a vat of 10 m × 25 m × 1 m. The thermochemical reaction started within 20 min, after which the workers stirred the mixture a few times with an excavator (Figure 1).

The reaction continued for at least 12 hr, becoming more explosive over time; it generated high temperature and intermittent firing, and profusely spread malodorous vapors. The warehouse had wide entrances on both sides, and all the window glass on the external wall had been removed, allowing the volatiles to spread throughout the factory (Figure 1). From May until November 2001, this process was carried out > 10 times each month. On 18 September 2001, we sampled the waste oils and solvents from the unslaked lime process and analyzed them by gas chromatography-mass selective detector (HP 5973 Series, Hewlett Packard, Wellesley, MA, USA). Because the process had been discontinued after the hepatitis outbreaks, we simulated this process on a smaller scale in the same building where this process had been conducted. Several chemicals, including dimethylformamide (DMF), dimethylacetamide (DMAc), and pyridine, were detected in some of the samples collected from a walk-through before and after the simulated process (Table 1).

### Clinical course of the cases

Case 1, a 51-year-old male, had been employed in the plant since 1986 and moved to the unslaked lime process with two other co-workers in July 2000. In April 2001, he had a single episode of chest pain. In early May, while he was hospitalized for the operation of sudden onset retinal detachment, his liver function was abnormal [aspartate aminotransferase (AST), 317 IU/L; alanine aminotransferase (ALT), 156 IU/L; alkaline phosphatase (ALP), 225 IU/L; total bilirubin, 2.2 mg/dL]. Ten days later his liver function was improved. He returned to work in the separator process in September, and then moved to the dry evaporative incinerator in October. However, his liver function worsened in November and peaked in December (AST, 482 IU/L; ALT, 507 IU/L). After stopping work, his liver function normalized.

Case 2 was a male, 35 years of age, who had started work in the evaporative concentration process in April 1994 with case 1. In late June 2001, he felt severely fatigued and began taking herbal medication 1 July to relieve the fatigue. However, his fatigue did not improve, and he developed insomnia, sweating, and jaundice 10 July. Case 2 was admitted to the hospital on 12 July after developing ascites with abnormal results of liver function (AST, 1,982 IU/L; ALT, 1,226 IU/L; ALP, 420 IU/L). He was discharged after remission of liver function on 30 August. After returning to work, he had a car accident and received injections of muscle relaxants and nonsteroidal anti-inflammatory drugs (NSAIDs) once a day for 8 days. He returned to work again on 24 September, but jaundice developed again on 1 October. On admission to the hospital at that time, his liver function worsened (AST, 737 IU/L; ALT, 432 IU/L; ALP, 541 IU/L; total bilirubin, 13.7 mg/dL). His condition worsened after admission, with severely increased liver enzymes and jaundice (AST, 193 IU/L; ALT, 159 IU/L; total bilirubin, 37.6 mg/dL). He died on 14 October 2001.

Case 3, a 41-year-old female, had been a repair and maintenance worker since 1993. In early July 2001, she felt severe fatigue and took two packs of herbal medicine given to her by case 2. On 14 July, her liver function test was normal. However, 1 week later her urine darkened and jaundice developed in her eyes. She was admitted to the hospital on 30 July with markedly increased liver enzymes (AST, 1,670 IU/L; ALT, 1,223 IU/L; ALP, 495 IU/L). Her condition improved steadily, and she was discharged on 19 September 2001. After returning to her job for 3 days, she felt so fatigued that she did not return to the workplace afterward.

Case 4 was a female, 53 years of age. She had been working in the sorting process since September 2000. She developed jaundice early in August 2001 and was admitted to the hospital on 24 August suffering from severe nausea. Her liver enzymes were markedly elevated (AST, 2,315 IU/L; ALT, 1,754 IU/L), bilirubin was elevated, and jaundice and mild ascites developed. After being moved to a university hospital, her condition improved gradually and liver function normalized on 12 December.

Case 5, a 26-year-old male, was employed in February 2001 and had been working in the dry evaporative incineration process. He was admitted to the hospital on 20 October 2001 due to jaundice and severely abnormal liver function results (AST, 1,336 IU/L; ALT, 1,368 IU/L; ALP, 198 IU/L). His condition improved, and he was discharged 8 November.

**Figure 2:**
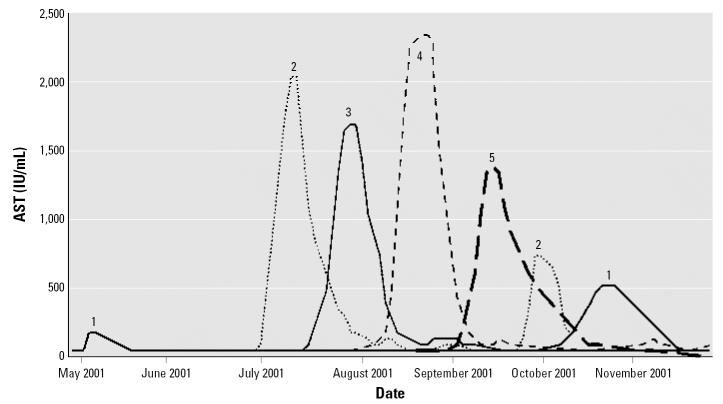
Clinical course for each case shown by AST level. Seven episodes of acute hepatic injury occurred in five workers from the plant. Case 1’s first episode occurred in May 2001, but the episode was limited only to laboratory findings. Most of the clinical episodes of acute hepatic injury occurred between July and September 2001. The second episodes of cases 1 and 2 developed after they returned to work.

Table 2 summarizes the employment and exposure history and clinical characteristics of cases 1–5. Figure 2 presents the clinical course of the cases as shown by AST concentration.

**Figure 3:**
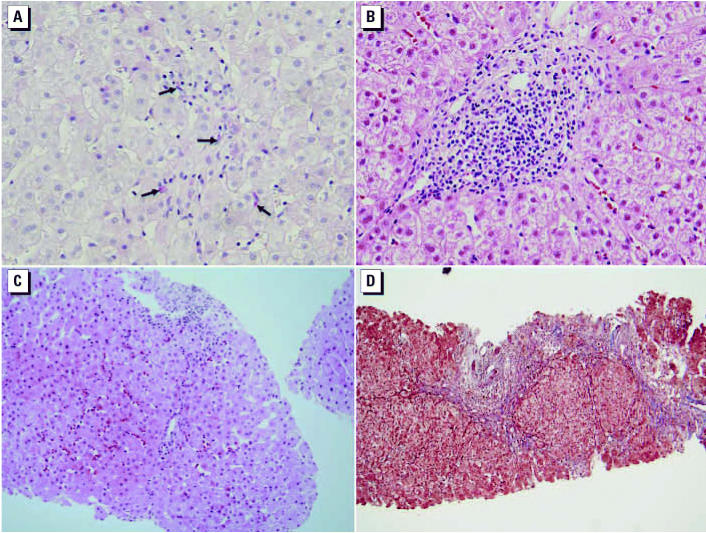
Pathologic findings of toxic hepatitis cases. (*A*) PAS-stained liver from case 5 showing spotty necrosis of hepatocytes and clumped Kupffer cells containing PAS-positive material (arrows); these find-ings are compatible with the remission stage of acute hepatitis and with toxic hepatitis (magnification, 400×). (*B*) Hematoxylin and eosin (H&E)–stained liver from case 1 showing portal tracts that are slightly enlarged and infiltrated with inflammatory cells (magnification, 400×). (*C*) H&E-stained liver from case 5 showing central to portal bridging necrosis (magnification, 200×). (*D*) Masson trichrome staining of liver from case 4 showing wide periportal necrosis extending into the portal-to-portal area, with regenerative nodules present (magnification, 100×).

Liver biopsy was performed on all but one case (case 2) in December 2001 (Figure 3). The pathologic findings showed diffuse spotty hepatocytic necrosis. The portal tracts were slightly enlarged, and inflammatory infiltration was present in all cases. Clumped Kupffer cells containing periodic acid-Schiff (PAS)-positive material were abundant, especially around the terminal hepatic venule (Figure 3A). These findings were compatible with the remission stage of acute hepatitis and also with toxic hepatitis. Wide periportal necrosis was also identified in cases 1 and 4 (Figure 3D), and necrosis was found in the central-to-portal or portal-to-portal region, with bridging necrosis in all cases (Figure 3C). In case 4, regenerative nodules were present, suggesting the development of cirrhosis. Cholestasis and fatty changes were also evident in cases 4 and 3, respectively.

Markers for hepatitis A (anti-HAV IgM) and B (HBsAg, HBeAg, anti-HBs, anti-HBc IgM) were negative in all cases. Anti-hepatitis C (anti-HCV) was positive in one case (case 1), but HCV was not detected on polymerase chain reaction (PCR).

This study was approved by the institutional review board of Dongguk University, Gyeongju Hospital. Written informed consent was received from all of the cases.

## Discussion

### Clinical evaluation

Seven episodes of hepatitis occurred in the five cases. Two of the patients suffered double episodes, the second of which occurred after the patient returned to work and received possible reexposure to the hepatotoxic agent(s). All of the episodes, except two, were very similar in their clinical course, with the duration of illness between 4 and 15 weeks (median, 7 weeks). The disease onset was highly concentrated within a narrow time period between May and August 2001.

The laboratory findings were quite similar for all of the episodes. The ratio of the relative increase of the measured values over the upper normal limit of ALT (40 IU/L) to that of ALP (120 IU/L) was > 5 in all cases, indicating that the hepatic injury was primarily hepatocellular rather than cholestatic. The hepatic lesions with bridging necrosis were also compatible with laboratory findings and clinical course. These characteristic values correspond with acute hepatocellular injury according to international criteria for diagnosis and classification (Danan and Benichou 1993).

None of the cases had hematologic, renal, or central nervous system symptoms or signs, but all of them had respiratory discomfort and dyspnea on exertion. None of the cases was positive for the immunologic indices such as antinuclear antibody or anti-smooth muscle antibody, and none had fever. Their clinical courses were acute hepatitis of less than 3-month duration.

Two episodes were exceptional. The first episode for case 1 was asymptomatic, and the liver enzyme level was not as elevated as the other five episodes. Also, the *R* value [(maximum ALT/upper limit of reference value of ALT for the illness) ÷ (maximum ALP/upper limit of reference value of ALP for the same period)] of 9.0 was lower than that of the five typical episodes (12.8–27.2). Case 2’s second episode was also different from the other typical episodes, with a lower *R* (7.1) and a relatively rapid increase in bilirubin concentration and hepatic failure. This episode more closely resembled fulminant hepatitis rather than typical acute hepatitis.

One of the most common causes of acute hepatic injury in Korea is viral hepatitis (Chun et al. 2002). In our cases, viral markers for HAV and HBV were all negative. HCV markers were positive in two cases. Case 1 was positive for anti-HCV, but viral RNA was negative by PCR. This absence of active viral RNA makes it unlikely that the acute hepatic injury was caused by HCV. In contrast, case 4 was positive in HCV-PCR but was negative for anti-HCV. Therefore, it is possible that HCV contributed to the development of acute hepatitis in this case. The prolonged course of case 4, compared with the other cases (15 weeks vs. 4–8 weeks), and the mild cirrhotic changes on pathology suggest the possibility of a mixed etiology for the illness of case 4.

When we applied the criteria for the diagnosis of drug-induced hepatic injury, our cases were compatible with toxic (or drug-induced) hepatic injury (Benichou 1990; Danan and Benichou 1993; Maria and Victorino 1997).

Severe destruction of liver cells with bridging necrosis was noted in all cases. However, in severe cases (cases 3 and 4), a wide area of necrosis was observed; this may indicate a change to subacute necrosis, which represents the possibility of transformation into chronic hepatitis. Actually, one case (case 4) already exhibited early cirrhotic change on pathology.

The role of herbal medication and hepato-toxic drugs in the development of acute hepatic injuries in two of the cases is of interest. Case 2 took herbal medications for 2 weeks and case 3 took only two doses. The label of the herbal medication indicated 28 components, at least one of which can be hepatotoxic (genistein, a phytoestrogen) (Hwang et al. 2001). However, the main reason case 2 took the herbal medication was for severe fatigue, which is related to the development of toxic hepatitis. In a case report of toxic hepatitis caused by this herb component, Hwang et al. (2001) described a cholestatic hepatitis that developed after the ingestion of a daily dose 10-fold higher than that of our case 4 for a period of 7 weeks. However, the strong similarity of the pattern of increase in the liver enzyme concentration and the *R* value, the similar clinical course of cases 2 and 3, and the short-term use of the herbal medication strongly suggests that the herbal medication was not a significant factor in the development of toxic hepatitis in these two cases.

Furthermore, contrary to cases 2 and 3, most reported cases of hepatitis related to herbal medications have been cholestatic hepatitis (Breen et al. 1986; Chitturi and Farrell 2000; Hwang et al. 2001; Langmead and Rampton 2001; Larrey and Pageaux 1995). These authors elucidated that the role of the herbal medication could be minor or, at best, an aggravating agent, rather than being a causative agent. In the case of case 3, she had only two doses of the herbal medication after her clinical manifestation had begun.

The NSAIDs taken by case 2 are also a potential candidate as the cause of his hepatitis. He received one ampule of 100 mg tolperisone HCl (a muscle relaxant) and diclofenac-β-dimethylaminoethanol (diclofenac) 90 mg/day for 8 days. Tolperisone has been reported to be hepatotoxic in animals but not in humans. In this case, the dosage given was much smaller than the reported toxic dosage. However, we could not completely exclude the possibility that it might have exaggerated his hepatitis during his second return to work.

Diclofenac has been reported to be a hepatotoxic compound (Bhogaraju et al. 1999; Breen et al. 1986; Helfgott et al. 1990; Iveson et al. 1990; Ouellette et al. 1991; Purcell et al. 1991; Ramakrishna and Viswanath 1994; Scully et al. 1993). From an evaluation of 180 cases of diclofenac hepatotoxicity, Banks et al. (1995) reported that the hepatotoxicity was usually asymptomatic, with a few mild symptoms. About 1–5 cases of diclofenac hepatotoxicity developed per 100,000 prescriptions, with the average latency of 3–12 weeks.

### Epidemiologic evaluation

Forty-eight workers (36 blue-collar workers and 12 office workers or drivers) have been working in this plant since 2001, and 6 workers, including two cases, retired after this outbreak of hepatitis. We evaluated liver function tests, ultrasonography, and immunologic markers of viral hepatitis in all employees except the three cases admitted to the hospital and three other absences. Two of them had mildly increased liver enzymes, and both had a previous history of abnormal liver function tests. One was suspected of having alcoholic liver disease and the other suffered from fatty liver due to being overweight. Based on ultrasonography, we suspected that 6 workers had fatty liver, which is one of the most common liver disorders among Korean workers (Cheong and Kim 1997). Another worker was suspected of having chronic hepatitis on the basis of ultrasonography. but did not have an abnormal liver function test. Of the 6 retired workers, 2 had chronic liver disease. A review of medical records showed that both had elevated liver enzyme levels from early 2001, both had positive HBV markers, and one was diagnosed with liver cirrhosis related to HBV and alcoholic liver disease. Therefore, they were not included in the cases. The incidence of toxic hepatitis was 10.4% in all of the workers and 13.9% in the production workers.

All of the cases developed within a 4-month period, from May to September 2001. Four of them occurred in a 2-month period, between mid-July and mid-September 2001. Even the recurrent cases of hepatitis occurred in this period. This phenomenon of heavy clustering over a narrow time span strongly suggests a possible association with exposure to toxic agents. The duration of illness in these cases was 4–15 weeks (median, 7 weeks).

**Figure 4:**
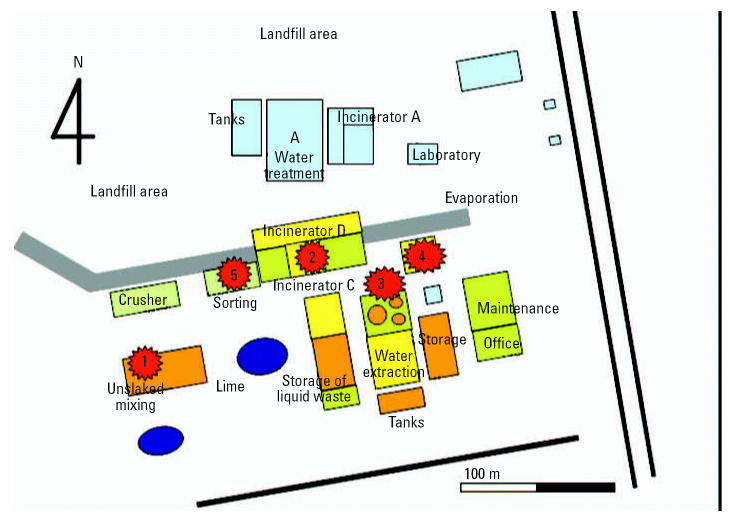
Diagram of the industrial waste plant showing the work areas of the hepatitis cases. Case 1 worked in unslaked lime processing. Workplaces of the other cases were northeast from the unslaked lime process warehouse. In this area, the seasonal wind from the southwest is limited to July.

All cases were production workers who had either participated in the unslaked lime process or worked nearby. In contrast, none of the workers who worked far from the unslaked lime process (e.g., laboratory, office, and waste water treatment staff) had hepatitis (Figure 4).

### Inference on the etiology

We suspect that the unslaked lime process is responsible for the development of this toxic hepatitis outbreak for the following reasons: *a*) during the 2 years before the outbreak, there were no significant changes in the amount and composition of the wastes or in the origin of the raw waste materials, except for the introduction of this unslaked lime process; *b*) no cases of hepatitis occurred before the introduction of the unslaked lime process; and *c*) simulation of the unslaked lime process showed that several hepatotoxic materials are generated in the waste and vapors. Investigations on the other possible sources, including drinking water, food supply, and specific process in each warehouse, did not suggest any other causative factors. The phenomenon of the highly time- and place-dependent clustering of the cases can be explained by the predominant wind direction. Annual climate data shows that the wind direction in July was from the southwest toward the northeast, the direction from the vapor source toward the victims; this explains the clustering of the cases between July and September.

Considering the clinical, epidemiologic, and pathologic characteristics, the derivable clues for causative agents of this outbreak are as follows: *a*) the main exposure occurred between June and August 2001; *b*) the agent should cause acute necrosis of liver parenchyma; *c*) an endogenous toxin is more relevant than an idiosyncratic reaction for the hepatotoxicity in these cases, given the relatively high incidence, although there is still a possibility of idiosyncrasy in view of the fact that cases 1 and 2 had eosinophilia and experienced a more severe, even fatal, relapse after reexposure; *d*) the zonal type of necrosis of hepatocytes was present, mainly zone I (periportal), but zone III (central vein) was also present in some of the cases; *e*) steatosis and cholestasis were only occasionally observed; *f*) the exposure route was suspected to be respiratory inhalation (neither oral intake nor skin absorption is plausible, considering the work processes and the raw material treated); *g*) the agent is probably a highly potent hepatotoxin with sufficiently high vapor pressure and toxic potency, which by inhalation, can cause toxic hepatitis in workers at least 100 m away from the source; *h*) the toxic activity of the agent should be specific to the liver without seriously affecting other organs or systemic effect; and *i*) the agent appears to have a relatively long latency (> 2 weeks), and repetitive or chronic exposure could also accelerate the toxic reaction.

Taking into account these inferences, we tried to match the clinical, pathologic, and toxicologic clues to the well-known hepatotoxic chemicals and those chemicals found in the samples from this industrial waste plant, such as DMF, DMAc, pyridine, and methylenedianiline (MDA) (Bastian 1984; Hall 1992; Kopelman 1966; McGill and Motto 1974; Tillmann et al. 1997; Williams et al. 1974). For each toxicant, we listed the toxic properties and its clinical characteristics, but none of them completely matched the criteria for the potential causative agent in the current cases.

DMAc and DMF, both solvents that can potentially cause acute toxic hepatitis (Baum and Suruda 1997; Choi et al. 2001; Kang et al. 1991; Kim et al. 1991; Marino et al. 1994; Spies et al. 1995), were detected in the environmental samples; such common solvents, which are used by many of the client industries of this plant, are likely to be the causative agents of this outbreak. Carbon tetrachloride is one of the strongest chemicals that can induce toxic hepatitis while showing hepatocellular necrosis with fatty change, but the clinical and pathologic characteristics we found do not correspond exactly with this agent, and it was not detected in our environmental samples. Pyridine, a hepatotoxin with its own peculiar odor, was also detected in the raw material samples (Baxter 1947; Carlson 1996; International Labor Office 1983; Pollock et al. 1943). Most of the hepatitis cases said that the vapor from the process was highly irritating; therefore, pyridine in the waste treated is a strong candidate. However, the environmental sampling was focused on the qualitative detection of the compound. Because the simulation of unslaked lime was performed on a much smaller scale than the true case and because the simulation was undertaken in November, the chemical composition may have varied significantly from that of June or August.

Another possibility is that the various chemicals detected in the analysis underwent an interaction among themselves, which synergistically raised their toxicity compared with the original material. Our cases showed relatively longer latency than usual toxic hepatitis; however, following the usual course of toxic hepatitis, there were no systemic symptoms or signs. Complex wastes have been evaluated for their toxicity (Meyer 1983; Simmons et al. 1988, 1994). It has been reported that the lowest dose of toxicity in a complex of several chemicals can be lower than that of any single chemical component, and latency can be longer (Dossing and Ranek 1984; Hodgson et al. 1989; Kimbroug 1983; Landrigan 1983; Neal 1983; Thilly et al. 1983). Simmons et al. (1988) suspected that toxic hepatitis could be caused by the synergistic interaction of various unknown chemicals generated during the unslaked lime process through thermochemical reactions.

In cases 2 and 3, herbal medicine and NSAIDs should be considered an aggravating factor—by synergistic or additive effect—on the liver damaged by hepatotoxic chemicals. However, we do not think they are the initiators. Cases 2 and 3 took the herbal medicine only after the severe fatigue had begun. Case 3 had only one dose of the herbal medicine after the symptom began. NSAIDs were given to case 2 after the recovery from the acute hepatitic episode. Intravenous injection of NSAIDs, together with resumed exposure could have triggered fulminant hepatitis.

## Conclusion

We conclude that this outbreak of toxic hepatitis developed as a result of exposure to the hepatotoxic materials generated during the treatment of industrial waste using unslaked lime. Between May and September 2001, five hepatitis cases occurred, including one death, due to fulminant hepatitis. The pathologic and epidemiologic findings clearly suggest that these cases of acute hepatitis were toxic hepatitis caused by occupational exposure rather than viral infection or medication, such as herbal medicine and NSAIDs. In environmental samples, we detected several hepatotoxic chemicals, including pyridine, DMF, DMAc, and MDA, in addition to solvents such as toluene, styrene, and xylene. Although we could not confirm the exact causative agent among them, the latency of the illness and other clinical characteristics strongly suggests that one of the chemicals, or the synergistic effect of the interaction among the complex components, evoked the hepatitis.

In general, the processes of treating industrial waste are conducted in confined areas. However, this plant had invented a new adaptation to improve the original unslaked lime process by introducing a thermochemical reaction. Waste oil or solvents are composed of many chemicals, with not only significant hepatotoxicity but also other organ toxicity. Therefore, these processes in the waste-treatment industry must be supervised and regulated under the direction of the public environmental protection authorities. Any arbitrary modification of such processes should be prohibited until the safety has been evaluated for any potential hazards to the environment and the workers.

## Figures and Tables

**Table 1:** Results of environmental monitoring in five locations and analysis of three bulk samples.

	Air samples (by location)									Bulk waste samples		
	EC		DI	ULP 1		ULP 2		ULP 3		B-ULP1	B-ULP2	A-ULP
Chemical	MSD (area %)	GC (ppm)	GC (ppm)	MSD (area %)	GC (ppm)	MSD (area %)	MSD (area %)	MSD (ppm)	GC (area %)	MSD (area %)	MSD (area %)	MSD (area %)
2-Butoxyethanol						4.20	1.5	17.88			1.5	17.88
MDA	0.63											
4-Methyl-2-pentanone	11.79			1.77		10.00				6.17		
Butyl acetate												
Cyclohexanone	1.93			2.05		1.66	2.09			3.59	2.09	
DMAc	2.17			0.87		0.86	0.25	3.52		1.52	0.25	3.52
DMF							1.75	2.35		1.04	1.75	2.35
1-Butanol							1.01				1.01	
Pyridine	3.60			1.77		1.93	26.07	2.08		1.65	26.07	2.08
Tetrahydrofurane	6.00			6.85		7.49	2.65	3.73		6.11	2.65	3.73
Toluene		0.015	0.041		8.996		—		9.127	1.39	—	
Xylene			0.026		7.646				8.052			
Trimethylbenzene	5.44											
1,4-Butanediol				4.58		5.48	30.33			28.37	30.33	
Pentadecane	1.71			2.54								
Tetradecane						1.05						
Total identified (%)	33.27			20.43		32.67	67.61	30.57		49.84	67.61	30.57

Abbreviations: DI, dry evaporative incinerator; EC, evaporative concentrator; GC, gas chromatography; MSD, mass selective detector; ULP, simulated unslaked lime process.

**Table 2:** Clinical characteristics of the toxic hepatitis cases.

Characteristic	Case 1	Case 2	Case 3	Case 4	Case 5
Age/sex	51 years/male	35 years/male	41 years/female	53 years/female	26 years/male
Department	Selection	Evaporator	Maintenance (welding)	Selection	Dry distillation incinerator
Job title	Sorting and treating waste liquids	Controlling evaporator	Welding and cleaning	Sorting waste	Controlling incinerator
Employment dates	20 December 1986–10 January 2001	18 April 1994	1 May 1993	1 October 2000	25 February 2001
Onset	April 2001	Late June 2001	Early July 2001	Early August 2001	Middle of 2001
Date of admission	1 May 2001	12 July 2001	30 July 2001	24 August 2001	19 September 2001
Duration of illness (weeks)^a^	2 (4)^b^	7 (9)	8 (10)	15	7
Viral hepatitis markers	HAV, HBV marker (−) anti-HCV (+) HCV PCR (−)	HAV, HBV marker (−) anti-HCV (−)	HAV, HBV marker (−) anti-HCV (−)	HAV, HBV marker (−) anti-HCV (−) HCV PCR (+)	HAV, HBV marker (−) anti-HCV (−)
Biopsy	Hepatitic damage Bridging necrosis Wide periportal necrosis	No biopsy	Hepatitic damage Bridging necrosis Fatty degeneration	Hepatitic damage Bridging necrosis Cirrhotic nodule Cholestasis	Hepatitic damage Bridging necrosis
					
*R*^c^ Comments	9.0/12.8^b^	27.2/7.1^b^ Took herbal medication and NSAIDs Patient died	23.1 Took herbal medication	24.5	19.7

Abbreviations: +, positive; −, negative.

a Time period between admission and the remission of the AST and ALT.

b 1st illness (2nd illness).

c (Maximum ALT/upper limit of reference value of ALT for the illness) ÷ (maximum ALP/upper limit of reference value of ALP for the same period).

## References

[b1-ehp0115-000107] Banks AT, Zimmerman HJ, Ishak KG, Harter JG (1995). Diclofenac-associated hepatotoxicity: analysis of 180 cases reported to the Food and Drug Administration as adverse reactions. Hepatology.

[b2-ehp0115-000107] Bastian PG (1984). Occupational hepatitis caused by methylenedianiline. Med J Austral.

[b3-ehp0115-000107] Baum SL, Suruda AJ (1997). Toxic hepatitis from dimethylacetamide. Int J Occup Environ Health.

[b4-ehp0115-000107] Baxter JH (1947). Hepatic and renal injury with calcium deposits and cirrhosis produced in rats by pyridine. Am J Pathol.

[b5-ehp0115-000107] Benichou C (1990). Criteria of drug-induced liver disorders. Report of an international consensus meeting. J Hepatol.

[b6-ehp0115-000107] Bhogaraju A, Nazeer S, Al-Baghdadi Y, Rahman M, Wrestler F, Patel N (1999). Diclofenac-associated hepatitis. South Med J.

[b7-ehp0115-000107] Breen EG, McNicholl J, Cosgrove E, McCabe J, Stevens FM (1986). Fatal hepatitis associated with diclofenac. Gut.

[b8-ehp0115-000107] Carlson GP (1996). Comparison of the effects of pyridine and its metabolites on rat liver and kidney. Toxicol Lett.

[b9-ehp0115-000107] Cheong HK, Kim JS (1997). A study on the prevalence and risk factors of liver dysfunction among the workers in chemical factories [in Korean]. Korean J Prev Med.

[b10-ehp0115-000107] Chitturi S, Farrell GC (2000). Herbal hepatotoxicity: an expanding but poorly defined problem. J Gastroenterol Hepatol.

[b11-ehp0115-000107] Choi TS, Woo KH, Kim JS, Park WS, Ham JH, Jung SJ (2001). Toxic hepatitis induced by occupational dimethylacetamide exposure [in Korean]. Korean J Occup Environ Med.

[b12-ehp0115-000107] Chun WJ, Yoon BG, Kim NI, Lee G, Yang CH, Lee CW (2002). A clinical study of patients with acute liver injury caused by herbal medication in Gyeongju area [in Korean]. Korean J Med.

[b13-ehp0115-000107] Danan G, Benichou C (1993). Causality assessment of adverse reactions to drugs—I. A novel method based on the conclusions of international consensus meetings: application to drug-induced liver injuries. J Clin Epidemiol.

[b14-ehp0115-000107] Dossing M, Ranek L (1984). Isolated liver damage in chemical workers. Br J Ind Med.

[b15-ehp0115-000107] Gehring PJ 1983. Pyridine, homologues and derivatives. In: Encyclopaedia of Occupational Health and Safety, Vol 2 (Parmeggiani L, ed). 3rd ed. Geneva:International Labour Office, 1810–1812.

[b16-ehp0115-000107] Hall AJ, Harrington JM, Waterhouse JA (1992). The Epping jaundice outbreak: a 24 year follow up. J Epidemiol Community Health.

[b17-ehp0115-000107] Helfgott SM, Sandberg-Cook J, Zakim D, Nestler J (1990). Diclofenac-associated hepatotoxicity. JAMA.

[b18-ehp0115-000107] Hodgson MJ, Van Thiel DH, Lauschus K, Karpf M (1989). Liver injury tests in hazardous waste workers: the role of obesity. J Occup Med.

[b19-ehp0115-000107] Hwang SH, Park JA, Jang YS, Lee KM, Lee DS, Ahn BM (2001). A case of acute cholestatic hepatitis caused by the seeds of psoralea – corylifolia [in Korean]. Korean J Hepatol.

[b20-ehp0115-000107] Iveson TJ, Ryley NG, Kelly PM, Trowell JM, McGee JO, Chapman RW (1990). Diclofenac associated hepatitis. J Hepatol.

[b21-ehp0115-000107] Kang SK, Jang JY, Rhee KY, Chung HK (1991). A study on the liver dysfunction due to dimethylformamide [in Korean]. Korean J Occup Environ Med.

[b22-ehp0115-000107] Kim SK, Lee SJ, Chung KC (1991). A suspicious case of dimethylformamide induced fulminant hepatitis in synthetic leather workers [in Korean]. Korean J Occup Environ Med.

[b23-ehp0115-000107] Kimbrough RD (1983). Determining exposure and biochemical effects in human population studies. Environ Health Perspect.

[b24-ehp0115-000107] Kopelman H, Robertson MH, Sanders PG, Ash I (1966). The Epping jaundice. BMJ.

[b25-ehp0115-000107] Landrigan PJ (1983). Epidemiologic approaches to persons with exposures to waste chemicals. Environ Health Perspect.

[b26-ehp0115-000107] Langmead L, Rampton DS (2001). Review article: herbal treatment in gastrointestinal and liver disease—benefits and dangers. Aliment Pharmacol Ther.

[b27-ehp0115-000107] Larrey D, Pageaux GP (1995). Hepatotoxicity of herbal remedies and mushrooms. Semin Liver Dis.

[b28-ehp0115-000107] Maria VA, Victorino RM (1997). Development and validation of a clinical scale for the diagnosis of drug-induced hepatitis. Hepatology.

[b29-ehp0115-000107] Marino G, Anastopoulos H, Woolf AD (1994). Toxicity associated with severe inhalational and dermal exposure to dimethylacetamide and 1,2-ethanediamine. J Occup Med.

[b30-ehp0115-000107] McGill DB, Motto JD (1974). An industrial outbreak of toxic hepatitis due to methylenedianiline. N Engl J Med.

[b31-ehp0115-000107] Meyer CR (1983). Liver dysfunction in residents exposed to leachate from a toxic waste dump. Environ Health Perspect.

[b32-ehp0115-000107] Neal RA (1983). Protocol for testing the toxicity of chemical mixtures. Environ Health Perspect.

[b33-ehp0115-000107] Ouellette GS, Slitzky BE, Gates JA, Lagarde S, West AB (1991). Reversible hepatitis associated with diclofenac. J Clin Gastroenterol.

[b34-ehp0115-000107] Pollock LJ, Finkelman I, Arieff AJ (1943). Toxicity of pyridine in man. Arch Int Med.

[b35-ehp0115-000107] Purcell P, Henry D, Melville G (1991). Diclofenac hepatitis. Gut.

[b36-ehp0115-000107] Ramakrishna B, Viswanath N (1994). Diclofenac-induced hepatitis: case report and literature review. Liver.

[b37-ehp0115-000107] Scully LJ, Clarke D, Barr RJ (1993). Diclofenac induced hepatitis: 3 cases with features of autoimmune chronic active hepatitis. Dig Dis Sci.

[b38-ehp0115-000107] Simmons JE, DeMarini DM, Berman E (1988). Lethality and hepatotoxicity of complex waste mixtures. Environ Res.

[b39-ehp0115-000107] Simmons JE, Yang RS, Svendsgaard DJ, Thompson MB, Seely JC, McDonald A (1994). Toxicology studies of a chemical mixture of 25 groundwater contaminants: hepatic and renal assessment, response to carbon tetrachloride challenge, and influence of treatment-induced water restriction. J Toxicol Environ Health.

[b40-ehp0115-000107] Spies GJ, Rhyne RH, Evans RA, Wetzel KE, Ragland DT, Turney HG (1995). Monitoring acrylic fiber workers for liver toxicity and exposure to dimethylacetamide: 1. Assessing exposure to dimethylacetamide by air and biological monitoring. J Occup Environ Med.

[b41-ehp0115-000107] Thilly WG, Longwell J, Andon BM (1983). General approach to the biological analysis of complex mixtures. Environ Health Perspect.

[b42-ehp0115-000107] Tillmann HL, van Pelt FN, Martz W, Luecke T, Welp H, Dorries F (1997). Accidental intoxication with methylene dianiline *p,p*′diaminodiphenylmethane: acute liver damage after presumed ecstasy consumption. J Toxicol Clin Toxicol.

[b43-ehp0115-000107] Williams SV, Bryan JA, Burk JR, Wolf FS (1974). Letter: Toxic hepatitis and methylenedianiline. N Engl J Med.

